# Size and Usage Patterns of Private TB Drug Markets in the High Burden Countries

**DOI:** 10.1371/journal.pone.0018964

**Published:** 2011-05-04

**Authors:** William A. Wells, Colin Fan Ge, Nitin Patel, Teresa Oh, Elizabeth Gardiner, Michael E. Kimerling

**Affiliations:** 1 Global Alliance for TB Drug Development, New York, New York, United States of America; 2 IMS Health, New York, New York, United States of America; 3 Bill and Melinda Gates Foundation, Seattle, Washington, United States of America; Kenya Medical Research Institute - Wellcome Trust Research Programme, Kenya

## Abstract

**Background:**

Tuberculosis (TB) control is considered primarily a public health concern, and private sector TB treatment has attracted less attention. Thus, the size and characteristics of private sector TB drug sales remain largely unknown.

**Methodology/Principal Findings:**

We used IMS Health data to analyze private TB drug consumption in 10 high burden countries (HBCs), after first mapping how well IMS data coverage overlapped with private markets. We defined private markets as any channels not used or influenced by national TB programs. Private markets in four countries – Pakistan, the Philippines, Indonesia and India – had the largest relative sales volumes; annually, they sold enough first line TB drugs to provide 65–117% of the respective countries' estimated annual incident cases with a standard 6–8 month regimen. First line drug volumes in five countries were predominantly fixed dose combinations (FDCs), but predominantly loose drugs in the other five. Across 10 countries, these drugs were available in 37 (loose drug) plus 74 (FDCs) distinct strengths. There were 54 distinct, significant first line manufacturers (range 2–11 per country), and most companies sold TB drugs in only a single study country. FDC markets were, however, more concentrated, with 4 companies capturing 69% of FDC volume across the ten countries. Among second line drugs, fluoroquinolones were widely available, with significant volumes used for TB in India, Pakistan and Indonesia. However, certain WHO-recommended drugs were not available and in general there were insufficient drug volumes to cover the majority of the expected burden of multidrug-resistant TB (MDR-TB).

**Conclusions/Significance:**

Private TB drug markets in several HBCs are substantial, stable, and complicated. This calls for appropriate policy and market responses, including expansion of Public-Private Mix (PPM) programs, greater reach, flexibility and appeal of public programs, regulatory and quality enforcement, and expansion of public MDR-TB treatment programs.

## Introduction

Tuberculosis (TB) remains a leading killer globally: despite notable treatment successes, it is associated with 1.7 million deaths every year [1], and there are more estimated cases each year based on population growth. Approximately 80% of the global TB burden is concentrated in 22 high burden countries (HBCs); 10 of these HBCs, covering 60% of the global burden [1], are included in this study. TB treatment has been considered primarily as a public health priority, because lengthy, supervised treatment is needed to maximize cure rates, reduce incidence, and minimize the development of resistance. Indeed, in the 10 HBCs under study here, 67% of estimated incident cases are at some stage detected and treated by national TB programs [1]. A significant amount of private sector TB treatment is, however, known to exist.

In the public sector, TB treatment policies are typically based on World Health Organization (WHO) recommendations [2], [3], and for first line treatment most countries adhere to a 6 month course denoted, in shorthand, as 2HRZE/4RH (2 months of isoniazid (H), rifampicin (R), pyrazinamide (Z) and ethambutol (E), followed by four months of isoniazid and rifampicin). Sales of standard dosages by the Global Drug Facility (GDF) reinforce these recommendations. For a given country, these treatment policies are usually decided centrally and are uniformly applied. Thus, estimating average treatment practices in the public sector is easier than in the private sector, where decision making is made at the healthcare provider or patient level.

We know, however, that private sector treatment is often inappropriate: in one study in India, 100 private doctors prescribed 80 different regimens [4], and a recent update showed no significant improvement [5]. Similarly, private providers in the Philippines were reported to use inappropriate regimens 89% of the time [6] and it is known that patients often run out of money and so cannot complete treatment in the private sector [7]. The World Health Organization (WHO) responded to these problems by introducing the concept of public-private mix (PPM), which links the private sector to public sector treatment norms [8], [9]; PPM has also benefited from use of the International Standards for Tuberculosis Care (ISTC) [10]. Unfortunately, however, the expansion of PPM in most settings has been relatively limited.

Studies of private sector behavior (as noted above) have generally been limited to small, localized samples that reveal diversity of practice but do not provide an overall sense of the major trends. Basic questions such as the relative size of the private market across HBCs, specific dosage strengths used, use of fixed dose combinations (FDCs) or loose pills, and leading manufacturers in this space have not been documented, despite the fact that the private markets in HBCs are not new and continue to serve, in certain countries, as major channels for treatment by patients. Our current analysis of TB drug sales data provides this missing overview of private market practices over time in a wide array of geographies.

Even less is known about possible private sector treatment of multidrug-resistant TB (MDR-TB). Despite an estimated burden of 440,000 new MDR-TB cases per year [1], public sector capacity for diagnosis and treatment is lagging: in 2008, only 7% of the estimated cases were detected and reported and less than one fifth of those reported cases were managed according to international guidelines [11]. The private sector may be stepping in to fill this gap, but data on this are lacking.

From a public health standpoint, understanding the private market is critical to ultimately improving overall outcomes in TB. In contrast to most National TB Programs (NTPs), the private markets operate in a decentralized and largely unregulated fashion, with few mechanisms in place to monitor and ensure evidence-informed prescribing and patient adherence to a TB regimen, creating risks for developing drug resistance [11]. To better understand how change can be effected in this sector, the global TB community first needs a baseline understanding of the TB market.

In a previous study, we focused on sizing the global market for TB drugs, including estimates of private TB drug market value for five high burden countries (HBCs) [12], [13]. Our new analysis covers five additional HBCs and provides, for the first time, a detailed definition and identification of private sector channels, the types of products being sold, their volumes, sales trends and the manufacturers producing these drugs. The results suggest a diverse, sizable and stable private TB drug market that will continue to affect treatment outcomes in both private and public sectors.

## Methods

IMS Health Inc. (IMS) tracks drug sales by recruiting various data sources (manufacturers, wholesalers, distributors, hospitals, pharmacies, other dispensing facilities, and healthcare providers), who submit prescription and drug sales information. The data collection strategy is different for each country based on the structure of the health system and the data sources available (see File S1), but in each case a set of channels (e.g., hospitals plus pharmacies plus non-pharmacy distributors) is used to construct a picture of the entire drug dispensing landscape. Results are validated by cross-checking with manufacturer data on specific products (see below).

Of the 22 high burden countries (HBCs) for drug-sensitive TB, only 11 are covered by current IMS data collection efforts. One of these 11 HBCs, Brazil, is thought to have no private market for TB drugs. We therefore focused on the remaining 10 HBCs (listed by 2009 HBC rank [14]): India (1); China (2); Indonesia (3); South Africa (5); Bangladesh (6); Pakistan (8); the Philippines (9); Russia (11); Viet Nam (12) and Thailand (18). Based on the estimated number of incident cases, these 10 countries include 60% of the estimated burden of TB [1]. IMS data were not available for other countries in sub-Saharan Africa including Nigeria (4), Ethiopia (7) and DR Congo (10).

For each of the study countries, we defined the “private market” as those channels that are not used or influenced by national TB programs (NTPs); this mirrors the revised definition for the scope of PPM [9] and is in many cases similar to the retail market considered, by drug companies, as distinct from the government tender market [13]. Government-funded treatment for particular sectors, such as the prisons, the military, or government railway workers, were considered more likely to follow NTP guidelines and thus were not included. In the remaining non-NTP channels, however, physicians and other health care providers are not obligated to comply with NTP guidelines or documentation requirements, and patients are often, but not always, responsible for treatment cost. Local IMS affiliates provided information on the major drug distribution channels and outlets in each study country; these channels were then classified as either (a) public sector; (b) private sector with IMS audit; or (c) private sector without IMS audit. Data were pulled from category (b) channels (see File S1 for details). Of note, in some countries certain hospitals are nominally public sector but have no link to the NTP (i.e., TB drug prescribing by health workers, and TB drug purchasing by both hospitals and patients, are independent of the NTP). For this study, such outlets were, to the extent possible, included as ‘private sector’ (see File S1).

In the channels defined as private, IMS coverage of outlets (i.e., number of outlets sampled divided by estimated total number of these outlets) ranged from 1–20%, with the exception of >80% coverage in South Africa and in one of the two relevant channels in Pakistan. Sampling reliability was assessed via the “precision index”, which is the percentage of all products (i.e., not restricted to TB drugs) for which the IMS prediction falls within +/−22.5% of manufacturer-supplied distribution (or sales) data. These precision indices ranged from 64–66% (for 3 study countries) to >80% (for the remaining 7 countries). In addition to this measure of clustering, the bias (average under- or over-estimation, when compared to manufacturer-reported figures) for these countries averaged −13% (range −19% to +2%, with the exception of Bangladesh (−25%) and Vietnam (−39%)).

In medical audits, a panel of 4,600 physicians (India) or 350–540 physicians (Indonesia, Pakistan, Philippines, South Africa, and Thailand) were asked to note the indication associated with each drug prescribing event over a period of one full week of prescribing. The physicians involved were internists or general physicians (30–50% of total), pulmonologists (2–5%) and other specialists. Such audits were not available for the four remaining study countries.

For second line drugs, we analyzed the overall market and their use specifically for TB. Volumes for TB were smaller relative to volumes for other indications, so analysis of the medical audit was more challenging. We only used medical audit data for which a 95% confidence interval was measurable and deviated < = 66% above or below the reported figures; the remaining figures were deemed unreliable.

For all countries, the drugs investigated were the four most common first line drugs (rifampin (R), isoniazid (H), pyrazinamide (Z), and ethambutol (E)), the 18 additional agents included in current WHO recommendations for second line treatment [15], and two additional fluoroquinolones (ciprofloxacin and gatifloxacin) that are not recommended but known to be used for TB. Volume data were reported in standard units, representing a single tablet, capsule, dose (for vials, ampoules or other liquid forms), or sachet. Drugs sold were used as a proxy for drugs used, as commercial considerations were expected to keep wastage to a minimum. Sales data were converted to US dollars at the time of data collection. Strengths were reported for > = 92% of volume for all except India (80%), South Africa (75%) and Bangladesh (54%), with most missing strengths being from 3- and 4-drug fixed dose combinations (FDCs).

Pricing data were adjusted for each country to the wholesaler acquisition level, using conversion factors calculated either at a margin mandated by the government (such as in South Africa) or using a large pool of products (such as in China). Prices for a first line regimen were calculated presuming a six month regimen and dosing of 3.5 tablets (average of the two most common weight bands) of the WHO-recommended 4-drug FDC RHZE (for 2 months) and 2-drug FDC RH (for 4 months), or their single drug equivalents. The same assumptions were used to convert first line drug volume data into “months of treatment” after first adjusting for the volume that had missing strengths. For the analogous calculation for fluoroquinolones, we assumed daily dosages of 800 mg (ofloxacin), 1000 mg (levofloxacin), or 400 mg (moxifloxacin or gatifloxacin), or twice daily dosage of 500 mg (ciprofloxacin).

Ethical approval was not required for the study as it relied on secondary data from a routine recording and reporting system.

## Results

### Alignment of IMS data channels with the private sector

As a first step in the analysis, we defined the “private sector” and its alignment with IMS data channels. The private sector was defined as encompassing all TB drugs that were being supplied outside of the policy or prescribing influence of NTPs (regardless of payer identity). This definition arguably made the most sense in terms of both working with IMS's data collection strategy (see below) and maximizing the policy relevance of the data. For some countries, this definition was simple: it included the retail market but excluded the bulk government tenders (which are often missing from IMS data anyway) [13], but for other countries the required subdivision of IMS channels was more complex (see File S1).

We rejected alternative definitions of the “private sector” such as “paid by private payer” or “supplied by for-profit provider”. IMS data do not include the identity of the drug payer (i.e., consumer, insurance company, or government), so measuring the volume of TB drugs paid for by consumers was judged impossible, at least with IMS data. Similarly, IMS data are not always sampled at the provider level. However, in all countries we could map a correspondence between certain IMS channels and our definition of the private sector. This strategy also measures the sector of most interest to policy makers: the channels that are outside of NTP policy oversight and thus have the most uncertain prescribing practices.

We evaluated three major areas of possible errors in aligning IMS channels and the NTP-independent private sector. First, our stated volumes could be underestimates as there are private sector channels in which IMS does not collect data, and there are also public sector channels that were excluded even though not all of their outlets (e.g., public hospitals in Viet Nam) collaborate with the relevant NTP. However, these missing channels were estimated to account for 20% (China) or < = 12% (all other study countries) of total pharmaceutical volume, based on comparisons between manufacturer and IMS survey data in each country. Second, the stated volumes could be an overestimate if NTP influence “contaminates” the choice of TB drugs in what we believe is the independent private sector. In China, for example, certain “designated hospitals” have established a collaboration with the NTP [16], although the extent may be limited as 150 of the 189 designated hospitals (as of 2009) were concentrated in 4 provinces with 17% of the country's population. Other PPM initiatives are limited in size, and most would not register in our analysis as they refer patients to the public sector for treatment [17] or use drugs supplied by the public sector [18], [19], [20], [21].

Finally, non-governmental organization (NGO) operations should not complicate our analysis. We consider it unlikely that a major NGO would be allowed to operate (by donors and national partners) if their TB regimens did not align with national and international norms; thus, NGO drugs should be omitted from our definition of the (non-NTP-influenced) private sector. This omission appears to happen in practice. In the study countries, Médecins sans Frontières, for example, either has no TB operations, or imports its drugs, procures via the public sector, or places orders directly with manufacturers (Carine Werder, pers. comm.). In Bangladesh, BRAC obtains TB drugs from the national TB program, which purchases from GDF (Md Akramul Islam, pers. comm.). None of these activities would be detected in our analysis. The exception may be NGO MDR-TB programs, some of which purchase second line drugs directly from the private sector, but most of these programs remain limited in size.

### Sizing of private, first line TB drug markets

Before considering first line drug volumes, we first checked the medical audits. The percentage of first-line TB drugs used for TB was > = 94% in Indonesia, Philippines and Pakistan, > = 89% in Thailand, > = 48% in South Africa (due primarily to classification of TB/HIV under “HIV”), and > = 67% in India (due primarily to additional sub-classifications of pulmonary disease not present on the IMS forms used in other countries). The two exceptions were for rifampicin in Pakistan (only 78% for TB, due mainly to use for brucellosis) and India (46%). As many of the “non-TB” uses appeared to misclassification of TB as other diseases, we made the simplifying assumption that all first line TB drugs in all 10 study countries were used for TB.

The ten study countries had private, first line TB drug markets that were either large (India, Indonesia, the Philippines, and Pakistan, with volumes of first line drugs sufficient to treat 65–117% of the estimated new TB cases in those countries in a given year with a full, daily 6–8 month regimen), medium (China, Russia and Thailand; 13–23%) or minimal (Bangladesh, South Africa and Vietnam; 3–7%) (Table 1). As a simplifying assumption, and purely for the purposes of illustration, we presumed that all patients received a full, daily course of the six-eight month WHO-recommended treatment regimen. Ethambutol usage was relatively higher in India, Philippines, Pakistan, and China, with figures being consistent with 20–40% of private sector usage involving 8 month regimens (2HRZE/6EH) in these countries. Alternatively, many other ethambutol-rich regimens may be in use. In total, private sector first line TB drugs in these 10 countries are sufficient to treat 66% of the countries' estimated incident cases, or 39% of the estimated incident cases worldwide. For 2008–9, they represented US$122 million of sales at wholesaler acquisition prices.

**Table 1:** Size and characteristics of private TB drug market.

Country	Incident cases (2008)	Coverage by first line, private sector drugs*	% change in volume 2004–9	% of private market that is loose drugs	Number of manufacturers with >3% of private first line market share	Fluoroquinolone coverage of incident MDR-TB cases#	Fluoroquinolone coverage of all incident cases&
India	1,982,628	117%	−3%	23%	6	41%	6.1%
Indonesia∧	429,730	116%	−5%	91%	6	12%	1.0%
Philippines	257,317	86%	−16%	16%	6		
Pakistan	409,392	65%	−7%	36%	4	13%	1.3%
China∧	1,301,322	23%	59%	98%	9		
Thailand∧	92,087	17%	−10%	94%	9		
Russia∧	150,898	13%	5%	100%	7		
Vietnam∧	174,593	7%	−28%	90%	11		
Bangladesh	359,671	7%	−51%	11%	2		
South Africa	476,732	3%	2%	34%	2		
Weighted average		66%	5%	52%			
Global Total	9,369,038						
10 country total, as % of global incidence	60%	39%					

*% of all incident cases that can be treated by first line drugs in private market (average across 4 first line drugs, assuming daily 6–8 month regimen). Data for this and other columns, unless noted, are for Q4 2008–Q3 2009.

∧ Denotes countries in which > = 90% of first line TB drugs in the private sector are loose.

# Assuming daily dosing for 18 month regimen, and no use for drug-sensitive TB.

& Assuming daily dosing for 6 month regimen, and no diagnosis of drug-resistant TB.

First line drug sales in the private market have been relatively stable during the period we studied (2004–2009). Change over the entire period was less than 10% in 5 HBCs; the exceptions were China (+59%), Thailand (−10%), Philippines (−16%), Viet Nam (−28%) and Bangladesh (−51%). Overall, 63% of the 10-country market is from India alone (whereas India represents only 35% of the 10-country overall TB burden); 85% is accounted for by India, China and Indonesia.

### FDC penetration of private markets

Each country's private market has shown a clear preference toward either FDCs or loose drugs: five countries predominantly use FDCs in the private market (India, Pakistan, Philippines, Bangladesh and South Africa have <36% use of loose drugs) while the other five countries typically use loose drugs (China, Indonesia, Russia, Thailand and Vietnam have > = 90% use of loose drugs) (Table 1). From 2004–2009, the largest volume trend between FDCs was a slight increase in the use of 4-drug FDCs (from 27% to 32% of all FDCs). In the FDC-dominant countries, 3-drug FDCs are either significant (26–37% of FDCs in India, Pakistan and Philippines) or almost absent (0–4% in Bangladesh and South Africa). Across all loose and FDC drugs, oral pills (tablets or capsules) account for 94% of volume.

### First line drugs are sold in a wide variety of strengths

**Figure 1:**
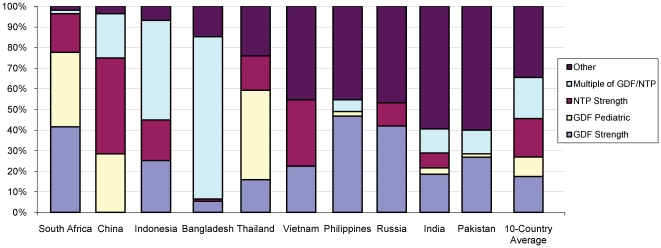
First line TB drug volumes broken down by strength, compared to GDF/NTP standards. First line drugs were classified as having strengths identical to those purchased by the Global Drug Facility (GDF) or recommended by the relevant National TB Program (NTP), or integer multiples of such strengths (e.g., twice as much or half as much). The remaining strengths (“other”; purple bars at top) constituted 35% of total volume. (Note: only products with known strengths were used in the calculation.)

**Figure 2:**
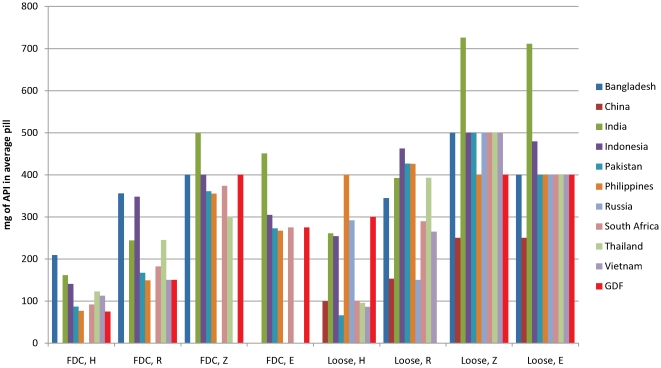
Strength variation for each first line drug. For each country and each active pharmaceutical ingredient (API), average strengths (weighted according to volume) were calculated and compared to those purchased by GDF.

For all four first-line drugs, a wide variety of strengths were sold. Diversity of drug strengths was high for both loose drugs (7–22 distinct dosage strengths per country; 37 in total) and FDCs (2–48 dosage strengths per country; 74 in total) (Table 2). Diversity was highest in India. Across all countries, non-standard strengths (neither identical to nor in a multiple of GDF or NTP recommendations) accounted for 35% of the volume of first line drugs with a known strength (Figure 1). However, there is notable clustering of the average strengths close to GDF-recommended dosages, particularly for loose Z and E (Figure 2), and the most popular 3- and 4-drug FDCs are at strengths sold by GDF. On average, tablets sold in China are of lower strengths and tablets sold in India are of higher strengths (Figure 2).

**Table 2:** Number of strength forms used for FDCs and loose drugs.*

Country	RH	HE	HZ	RHE	RHZ	RHZE	Total FDC dosages in country	R	H	Z	E	Total loose dosages in country
India	15	2		8	15	8	**48**	5	2	7	8	**22**
Philippines	7	4		3	3	2	**19**	6	5	2	2	**15**
Pakistan	5	3		3	4	2	**17**	5	2	2	2	**11**
Indonesia	3	6			1	1	**11**	5	4	3	2	**14**
Bangladesh	5				4	1	**10**	3	2	1	1	**7**
Vietnam	3	1		1	1	1	**7**	3	2	1	1	**7**
South Africa	4				1	1	**6**	4	1	1	1	**7**
Thailand	2				1	1	**4**	4	2	1	2	**9**
Russia	1	1	1			1	**4**	4	3	1	1	**9**
China	1				1		**2**	8	3	1	1	**13**
**10-country total**	**20**	**13**	**1**	**11**	**18**	**11**	**74**	**11**	**9**	**8**	**9**	**37**

*Products with unknown strengths were not included in this analysis.

### Private first line drug prices are usually higher than GDF's list price

**Figure 3:**
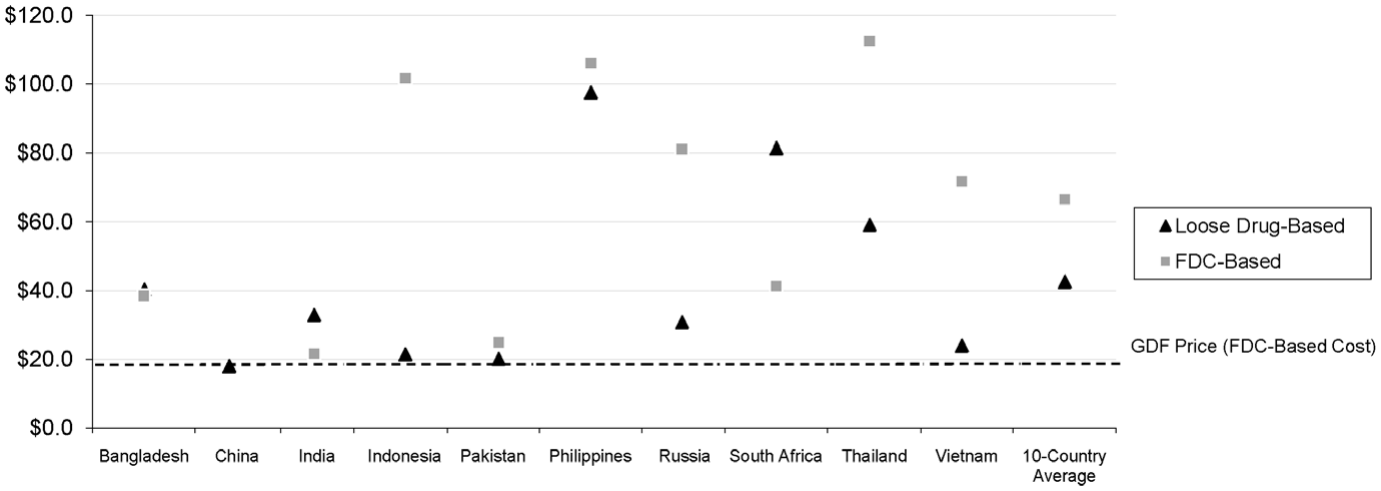
Prices of loose drug- and FDC-based 6 month regimens. Average prices per mg (for loose drugs) and for 4- and 2-drug FDCs (for FDCs) were used to calculate the average cost of a daily, 6 month 2HRZE/4HR regimen.

Not surprisingly, the average drug cost in the private market is often higher than the procurement prices listed by GDF (∼US$25 per 6 month course), with wholesaler acquisition prices of loose and/or FDC-based regimens over US$50 in Indonesia, Philippines, Russia, South Africa, Thailand and Vietnam (Figure 3). The lowest price was in China, which uses provincial level bidding for both NTP and non-NTP facilities. In countries with relatively little FDC use, the FDC-based regimens are significantly more expensive than loose-drug-based regimens; this rule does not hold in the other 5 countries.

### The first line market is fragmented overall, but the FDC market is more concentrated

**Figure 4:**
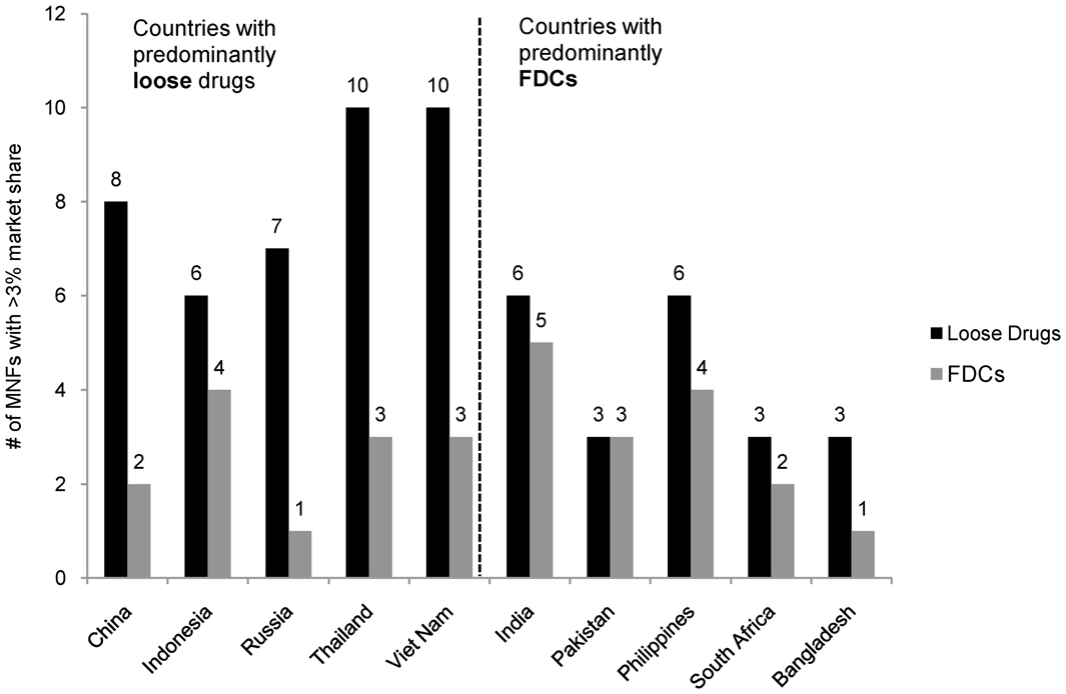
Number of manufacturers with first line FDC or loose drug market share greater than 3% (by volume, 2008–2009). Compared to countries with predominantly FDCs, countries with predominantly loose drugs have more manufacturers with >3% first line market share of either loose drugs (40 vs 17) or all first line drugs (40 vs 16). Numbers in figure legend are not a simple summation of numbers in figure as some companies are present in multiple countries.

There were 54 distinct first line manufacturers with >3% market share in at least one country (range 2–11 per country). Lupin, Wyeth and Sandoz are the only three of the top 12 first line manufacturers that sell products in the private markets of more than one study country: they cover two, two and eight of the study countries, respectively. Thus, most of the private markets are fragmented and include a large number of domestic manufacturers. However the FDC market is relatively more concentrated than the loose drug market, likely due to the challenges associated with producing FDCs. As a result, the five countries where FDCs are dominant have only 16 unique manufacturers with >3% total share, compared to 40 across the remaining five countries. Figure 4 presents a country by country comparison.

The top 10 manufacturers of loose drugs account for 62% of the 10-country loose drug market. For FDCs, the top 10 and top 4 FDC manufacturers account for 87% and 69%, respectively, of the FDC market. Three of the top four FDC manufacturers – Lupin, Macleods and Sandoz – produce FDCs that are pre-qualified by WHO. Although these companies produce 56% of the FDCs in the private markets in the study countries, only 7% of that 56% is in dosage forms that are prequalified (and likely even less would be from the prequalified manufacturing sites).

### The 2nd line market across all indications lacks certain drugs

The market volume for second line drugs (used for both TB and non-TB indications) has been growing (at 5% or more per year) and is dominated by India (63%) and China (17%). Most countries lack any significant (>2000 standard units) private sector sales volumes of capreomycin, clofazimine, and most of the oral bacteriostatic agents (Table 3). On average, each of the ten countries lack significant volume of 4.8 out of the 17 recommended second line drugs (range 0–8), but seven of the countries have at least one drug available in each of the categories. Volume is, however, concentrated in certain drugs: across all indications, fluoroquinolones and amoxicillin/clavulanate account for 96% of volume of second line drugs (43% of the total is ciprofloxacin alone); the only other drugs with >1% share are amikacin and clarithromycin.

**Table 3:** Certain 2nd line drugs (for all indications; quarter 4, 2008–quarter 3, 2009) are not available in all countries.*

Category	API	Bangladesh	China	India	Indonesia	Pakistan	Philippines	Russia	SA	Thailand	Vietnam
**Injectable agents**	Streptomycin	Low									
	Capreomycin	Absent			Absent	Absent	Absent	Low	Low	Absent	Absent
	Amikacin										
	Kanamycin						Absent				
**Fluoroquinolones**	Ofloxacin										
	Moxifloxacin										
	Levofloxacin										
**Oral bacteriostatic agent**	Protionamide	Absent			Absent	Absent	Absent		Absent	Absent	
	Ethionamide	Absent	Absent		Absent		Absent	Low			
	Terizidone	Absent	Absent		Absent	Absent	Absent	Absent		Absent	Absent
	Cycloserine	Absent	Absent		Absent		Absent		Absent		Absent
	Aminosalicylic Acid	Absent			Absent		Absent		Absent		
**Agents with unclear role and non-recommended quinolones**	Gatifloxacin							Absent			
	Ciprofloxacin										
	Clarithromycin										
	Cilastatin+Imipenem										
	Amoxicillin+Clavulanic Acid										
	Linezolid							Low			Low
	Clofazimine	Absent	Absent		Absent	Absent	Absent	Absent	Absent		Absent

*“Absent” means a product was not present in the private sector sales data of a particular country between quarter 4, 2008 and quarter 3, 2009; “Low” means that the product's volume was less than 2,000 standard units.

### Fluoroquinolone volumes and coverage of the MDR-TB burden

Six countries have medical audits, allowing assessment of second line drug volumes used for TB. Across all second line drugs for TB in these countries, India is even more dominant, accounting for 93.5% of the total volume (whereas India represents only 66% of the estimated 6-country overall MDR-TB burden).

With certain drugs in certain countries, very small volumes for TB rendered the medical audit unreliable. However, there were three countries in which at least the three fluoroquinolones most commonly used for TB (ciprofloxacin, ofloxacin, and levofloxacin) had measurable error margins after the medical audit. Actual dosaging practices may vary but, for illustrative purposes, the amount of measurable fluoroquinonolones used for TB could supply the daily dosages for an entire 18-month WHO-recommended MDR-TB treatment regimen for 54%, 15% or 11% of total estimated incident MDR-TB patients [22] in India, Indonesia, and Pakistan, respectively. Alternatively, use of these drugs as a daily addition to six month first line regimens (a non-recommended but known practice) would result in coverage of 6%, 1.3% and 1% of estimated incident drug-sensitive TB patients. Ciprofloxacin, a fluoroquinolone not recommended for TB [15], made up 29%, 38% and 38% of all measurable fluoroquinolones for TB in these countries.

Extending our analysis beyond the fluoroquinolones, we note that second line drug usage for TB is concentrated among very few drugs. In India, Indonesia, Pakistan and the Philippines, the four countries with the largest drug volumes and most reliable medical audits, fluoroquinolones and streptomycin account for 74% and 14%, respectively, of 2nd line volume for TB. (Both drugs were counted here as second line drugs.) This leaves only 1% of volume for the other, preferred injectables such as kanamycin, capreomycin and amikacin, and 9% for the bacteriostatic agents. Thus, we conclude that the percentage of the MDR-TB burden covered by complete, rational MDR-TB regimens from the private sector is likely to be small, with even an optimistic estimate being ∼8-fold lower than the fluoroquinolone numbers cited above.

## Discussion

Private markets are an important part of the TB treatment landscape. This study represents, to our knowledge, the first detailed overview of the private TB drug market across countries with the majority of the world's TB burden. Whereas our previous market study focused on capturing all TB drug value, regardless of source, we now place an emphasis on defining the private sector and providing an analysis of its characteristics in terms of both products and manufacturers.

Based on the data in this study, the private TB drug market is stable, sizable in several HBCs, and demonstrates extensive variation in drug manufacturing and utilization patterns. The variation in first line dosage strengths and the low volumes or lack of availability of a number of second line agents suggest that both first line and second line treatment in the private sector diverge significantly from international and national guidelines. This, combined with the previous evidence of variable and incomplete treatment in the private sector, raises a considerable risk of treatment failure and the development of drug resistance, and strengthens the evidence supporting bold action in engaging the private sector.

In the study countries, the number of incident TB patients treated in public programs [14] and the number potentially treated with private sector drugs (based on this study) are similar (67% and 66% of incident cases, respectively). In a recent survey [3], TB stakeholders provided estimates of private market size in the 22 HBCs [23]. The relative ranking between countries from this exercise was similar to that from the IMS drug usage data. However, based on the IMS data, the absolute private market sizes are substantially greater than those estimated by stakeholders, likely due to repeated treatments in private and public sectors (and possibly unrecognized burdens of TB and treatment of pneumonia with TB drugs). The large size of certain private first line TB drug markets is consistent with some previous reports, e.g., that in India 86% of patients first sought help in the private sector [24], and that private practitioners provide overly lengthy regimens [5].

Private market size has remained stable despite the efforts by NTPs in many countries to increase their share of the TB treatment burden, and despite some indications that patient perceptions of public sector TB treatment are changing [7]. This suggests that the non-NTP providers in several HBCs are fulfilling a continued need in the eyes of consumers, who choose to use the private market even though free products are available through their respective NTPs.

A variety of steps can be taken to prevent or limit irrational TB drug dispensing in the private sector [11]. Regulations can be used to restrict TB medications to the public sector (e.g., in Brazil); even in this scenario, an efficient referral system is needed to reduce diagnostic delays for patients first accessing the private sector. Of note, our data show private sector TB drug volumes even in a country (Russia) thought by stakeholders to have only public sector TB drugs [23].

In countries with existing private TB drug markets, or for TB drugs that have other major indications (e.g., fluoroquinolones), a strictly regulatory approach may not be realistic. Other interventions are possible, however. Public sector programs can be made more extensive, appealing and flexible to reduce consumers' private sector preference [25], and innovative and strengthened PPM collaboration can be used to bring public sector treatment norms to private sector providers. The size of these responses should reflect the scale of the problem as identified here.

For PPM, subsidized drugs appear to be an important component for success in many settings [18], [26], although some interventions concentrate primarily on advocacy around treatment norms [27]; of note, the impact of this latter intervention would not be visible in our data. Increasing implementation of the International Standards for Tuberculosis Care (ISTC) [10] via professional associations, with a greater emphasis (or a special campaign) on the standard regimens and dosages recommended, could also help make private TB treatment more rational.

One reason for concern about the private market is the variation in dosing practices, which has been documented previously at the provider level [4], [5], [6]. With our data, we cannot determine exact prescribing behaviors or the rationale behind those behaviors, but we have obtained a comprehensive view of the large numbers of dosages available to providers. With the option of daily or intermittent dosing, the use of weight bands, an old and incomplete evidence base, and variable practices between countries, it becomes challenging to distinguish rational from non-rational use. Even with standardized regimens, dosage errors can be common [28], and dosage profusion only raises the likelihood that private providers may make mistakes or that their patients will be confused. Thus, the private sector is keeping alive the confusion that existed previously in the public sector. Just as WHO and the GDF helped to rationalize dosing in the public sector [29], so regulators may be able to use market authorization to rationalize private markets. Some guidance from WHO on this issue, geared specifically to an audience of national regulatory authorities, would seem appropriate. Manufacturers may also be persuaded via provider demand to make only the strengths recommended by WHO or the NTPs.

The fragmentation of the global TB drug market has been noted previously [12]. We found, however, that such fragmentation is less pronounced in the FDC market, probably because FDCs are newer and more challenging to manufacture. Action at just four manufacturers (Lupin, Macleods, Wyeth and Sandoz) – e.g., to limit production to rational dosages made with high quality assurance – could reduce drug quality concerns for 69% of the private sector FDCs in these 10 countries. In addition, manufacturers could promote more uniformity of treatment via standardization of information in package inserts and roll-out of better provider educational materials.

In countries where FDCs have come to dominate the private sector, their prices have fallen to equal or even undercut the prices for loose drugs, an important learning for countries considering a public sector switch to FDCs. Indeed, the number of manufacturers is sufficient to create competition in both FDC and loose drug markets, and this competition may encourage manufacturers to differentiate their products by creating large numbers of strength variants. Confirmation of this hypothesis would require manufacturer interviews.

Others have noted the poor availability of certain second line drugs, notably capreomycin and clofazimine, despite their relative desirability within their respective classes of MDR-TB drugs [30]. Our data on second-line drugs, though incomplete, are consistent with these statements. Several WHO-recommended drugs are not sold, and fluoroquinolones (and, to a lesser extent, streptomycin) dominate the landscape. Thus, it would be challenging for private providers to construct a full MDR-TB regimen in the private sector, even if they had the requisite training and laboratory facilities. However, the presence of many second line drugs suggests that drug supplies may be available for possible scale-up of MDR-TB treatment, either in the public or private sector. In the largest private TB drug market, India, all second line drugs are available, and volumes may expand greatly when rapid point-of-care tests such as GeneXpert® MTB/Rif are scaled up in the private sector.

Significant fluoroquinolone usage for TB does exist in several countries, although given the much smaller volumes of other second line agents, the fluoroquinolones are likely being used as monotherapy or as an add-on to first-line therapy rather than as part of a designated second-line therapy. (Based on the past history of TB regimens, private providers may also consider streptomycin, even more than fluoroquinolones, as a first line drug.) Thus, it seems that most MDR-TB patients are not receiving sufficient MDR-TB treatment in either the public [22] or private sector; the vast majority of the estimated 440,000 new MDR-TB cases each year [1] are likely not diagnosed or treated with rational MDR-TB regimens. The limited private sector usage of most second line drugs may be slowing the emergence of additional second line drug resistance, although the finding of significant fluoroquinolone usage for both TB and non-TB indications is consistent with the increasing incidence of fluoroquinolone-resistant TB in certain countries [31]. As the demand for both fluoroquinolones and other second line drugs expands in the private sector, regulatory and oversight approaches will become all the more important to protect both lives and drugs.

A secondary goal of this study was to evaluate the robustness of IMS data coverage for the TB drug markets in HBCs. IMS databases are a commercial product, and sampling is conducted if and when the effort and cost required can be supported by the commercial value of the resulting data. As a result, data coverage is incomplete in lower income markets. These limitations include a complete lack of sampling (in 11 of the HBCs), less complete sampling than in higher income countries (for the remaining 11 HBCs), no medical audit (in 4 of the remaining HBCs), smaller medical audits, and certain additional data gaps.

Not surprisingly, IMS coverage is relatively low in the covered HBCs (sampling 1–20% of all outlets, compared to 72% of wholesalers in the USA or 94% of hospital beds in the UK, for example). However, comparison with manufacturer data indicates an acceptable level of accuracy (see Methods). Certain channels are missed completely but relatively minor (see Results and File S1).

Medical audits, when available, proved reliable for first line drugs due to large volumes and few alternative indications. However, they were insufficient to provide reliable TB usage estimates for a number of the second line drugs in countries with less private sector TB treatment. For example, in some countries data on the usage of fluoroquinolones for TB was swamped by their usage for other indications, often in other formulations.

Every effort was made to exclude any private sector channels that were under the influence of the relevant NTP, and any challenges were judged to be manageable (see Results and File S1). We also considered that data collection may be biased away from outlets that serve lower income areas and clients, thus leading to an under-estimate of TB drug volume. (The reverse bias is also possible but arguably less likely.) This issue is a greater concern when measuring TB drug consumption because of the extreme concentration of TB in low income communities. It was not possible to evaluate this possibility, or its potential impact, although we note that IMS data in large countries are collected independently in multiple regions to minimize geographical bias. In the future, it may be worth embedding a simple, one-off income or poverty survey in the IMS data collection mechanism to determine whether any anti-poor bias exists.

On balance, these data issues suggest that under-estimation is more likely than overestimation, and that the major conclusions of this study are not likely to be affected by any of the data uncertainties. IMS is increasing efforts to strengthen data collection in India given the country's growing economic position; however, sample size in rural areas, where many TB patients are treated in the private sector, may continue to be limited. For many other HBCs, a lack of financial incentive for IMS, combined with the complexity of the various supply chains, may continue to be a barrier to data-strengthening efforts. A recent public-private partnership between the Medicines for Malaria Venture (MMV) and IMS is now, however, seeking to fill this gap in Sub-Saharan Africa (Renia Coghlan, pers. comm.).

### Conclusion

The 10 HBCs varied significantly in the size of their private markets for TB drugs. Particularly large and stable private sectors for first line drugs were present in 4 HBCs, where carefully documented public sector treatment numbers may obscure the amount of private sector treatment occurring beforehand. In considering interventions to address private sector TB treatment, the size of the response should be commensurate with the size of the problem. Greater government and international support for implementing PPM, expanding the reach of public programs, improving regulatory oversight for both marketing approvals and quality assurance, and expanding public sector MDR-TB diagnosis and treatment could have a great impact in strengthening TB treatment outcomes in the entire health sector.

## Supporting Information

File S1 The supporting information describes the major TB drug channels in each study country, the channels audited by IMS, and how the IMS audit and private drug channels overlap.
(DOC)
