# Supplementary material for: Size and Usage Patterns of Private TB Drug Markets in the High Burden Countries
Source: PLoS One. 2011 May 4;6(5):e18964. doi: 10.1371/journal.pone.0018964 (PMC3087727; doi:10.1371/journal.pone.0018964)
Supplement: File S1 — The supporting information describes the major TB drug channels in each study country, the channels audited by IMS, and how the IMS audit and private drug channels overlap. (DOC) [file pone.0018964.s001.doc]

**Supporting Information File S1**

**IMS Audit Details**

The following paragraphs describe the major TB drug channels in each study country, the channels audited by IMS, and how the IMS audit and private drug channels overlap. Note that IMS data were not adjusted upwards if the audit did not cover all relevant distribution channels. The drug coverage estimates below are based on assessing usage of all pharmaceuticals, not just TB drugs.

Bangladesh

In Bangladesh, there are three major channels where patients can obtain TB therapies—NTP sponsored chest disease clinics and district hospitals; pharmacies; and private clinics and practitioners’ offices. The IMS audit in Bangladesh covered pharmacies, where 92% of all pharmaceuticals in the private sector are thought to flow, but not NTP clinics or private clinics. There were no major concerns about under- or over-estimation.

China

In China, there are four major channels where patients can obtain TB therapies—NTP (TB dispensaries); hospitals ≥100 beds (TB or non-TB hospitals); hospitals <100 beds (TB or non-TB hospitals); and drug stores. The IMS audit in China covered hospitals with ≥100 beds, where 80% of drugs are thought to flow, but not hospitals with less than 100 beds or drug stores. There may be an under-estimation of the data in China (~20%) due to lack of coverage of small hospitals but also an over-estimation (~14%) due to recruitment of non-NTP hospitals by the NTP (see below).

The majority of public sector TB treatment in China occurs in TB dispensaries. There are, however, two important categories of hospitals where TB treatment is officially allowed:

- “Designated hospitals”: these hospitals are designated by CDC and provide NTP-funded drugs to patients. These hospitals should be considered as representing the public sector.
- “TB hospitals”: these hospitals (e.g., Beijing Chest Hospital) provide TB treatment but do not have a collaboration with the NTP and thus should be considered as the private sector.

Given the setup of the TB care system in China, the hospital data from IMS may include sales from both “designated hospitals” and “TB hospitals”. Therefore, it is important to estimate the sales from “designated hospitals”. In other words, because the public sector increasingly recruits non-NTP hospitals, this may lead us to overestimate the size of the true private market. We believe that the level of overestimation will be small since only 189 hospitals nationwide (out of ~8000 hospitals with ≥100 beds in total) have been recruited by the public sector. Because IMS data in China are not broken down by province or hospital type, we used province populations to estimate the maximum weight of sales from “designated hospitals”. As of early 2009, there were 189 designated hospitals, and 150 were concentrated in four provinces: Zhejiang, Shanghai, Jiangxi and Jiangsu (Cornelia Hennig, pers. comm.). Even if all sales in these four provinces were considered to be public sector, the population from these provinces accounts for only ~14% of the overall population in China; therefore, we estimate that at most ~14% of the sales in IMS’ hospital data might be considered public sector.

India

In India, there are five major channels where patients can obtain TB therapies—RNTCP affiliated facilities; retail pharmacies; private hospitals and clinics; dispensing practitioners; and hospitals, institutions and clinics that purchase directly from manufacturers. The IMS audit in India covered retail pharmacies, private hospitals and clinics, and dispensing practitioners, where 95% of non-RNTCP drugs are thought to flow, but not private hospitals, and institutions and clinics that purchase directly from manufacturers. There were no major concerns about under- or over-estimation.

Indonesia

In Indonesia, there are five major channels where patients can obtain TB therapies—NTP sponsored microscopy and independent health centers (PUSKESMAS); pharmacies; drug stores; hospitals; and other dispensing facilities. The IMS audit in Indonesia covered pharmacies, drug stores, hospitals, and other dispensing facilities, where 100% of private sector drugs are thought to flow. IMS coverage of “other dispensing facilities” (e.g., unlicensed drug stores, supermarkets, and dispensing physicians) is new since our previous survey of drugs [1], thus increasing the accuracy of the estimate.

Most government hospitals should generally be considered as being outside the influence of the NTP in the area of provision of medicine (and thus, for this study, effectively private sector). However, the NTP is reported to have recruited 30-40% of these hospitals nationwide for participation in DOTS (which is reflected in a scale up from 27 to 51% DOTS case detection in the past 5 years). Many of these recruited hospitals receive free drugs from the NTP; these free drugs will not be detected by the IMS audit, which is invoice based, so they do not create any complication for our study. Certain facilities may, however, have a mixture of these free drugs from the NTP (thus resulting in NTP influence over their prescribing behavior) and additional TB drugs purchased using the facilities’ own funding or money from social insurance schemes. It is not possible to tease apart this complexity with the current data collection methodology. Finally, a similar possibility for mixed drug inputs arises at clinics run by NGOs and faith-based organizations, and at private hospitals and private doctors’ offices that have agreed to follow NTP reporting. However, in this latter category only ~5% of private facilities are estimated to report to, and receive drugs from, the NTP.

Pakistan

In Pakistan, there are four major channels where patients can obtain TB therapies – NTP-sponsored district hospitals, TB sanatoriums and TB centers; retail pharmacies; wholesalers distributing to non-pharmacy (unlicensed) chemists and grocery stores; and private hospitals, clinics, and general practitioners. The IMS audit in Pakistan covered retail pharmacies and wholesalers, where 79% of public and private drugs are thought to flow, but not private hospitals and clinics. There may be a slight under-estimation since private hospitals (~12% of total drug flows) were not a part of the audit.

Philippines

In the Philippines, there are four major channels where patients can obtain TB therapies – government health centers integrated with the NTP; hospitals (both public and private); pharmacies (chain or independent); and private clinics and dispensing doctors' offices. The IMS audit in the Philippines covered hospitals and pharmacies, where 88% of non-NTP drugs are thought to flow, but not private clinics, so there may be a slight under-estimation. Although public hospitals were included, TB drugs are supplied to public hospitals free of charge, so these drugs would not be captured by the invoice-based IMS audit. Relative to other countries, the audit in the Philippines relies more on wholesaler data, but these data are screened so that only sales to hospitals and pharmacies are included (i.e., sales to local government entities are excluded). In addition, some under-estimation is possible as the wholesalers may not cover the sales from all manufacturers, especially for certain unbranded generics.

Russian Federation

In Russia, there are three major channels that supply TB therapies—the NTP-influenced city/district hospitals or local dispensaries; departmental purchases (mainly prison and military); and retail pharmacies. We included the IMS audit in Russia that covered retail pharmacies, where 80% of drugs sold in hospitals and pharmacies are thought to flow (the remaining 20% are in the NTP-influenced city/district hospitals or local dispensaries). Sales from hospitals, including private hospitals, were excluded, as the relevant IMS data do not differentiate between public and private hospitals, and only an estimated 2% of hospitals are private.

South Africa

In South Africa, there are two major channels for the flow of TB drugs – public sector provincial depots that supply public clinics, hospitals and health centers; and private sector distributors and wholesalers who supply employers and private hospitals and clinics. The IMS audit in South Africa covered distributors, wholesalers, and employers, where 95% of drugs are thought to flow. There were no major concerns about under- or over-estimation.

Thailand

In Thailand, there are four major channels where patients can obtain TB therapies— government health centers; hospitals (both public and private); pharmacies; and private health centers, clinics and convenience stores. The IMS audit in Thailand covered hospitals and pharmacies, where 92% of drugs are thought to flow, but not private clinics. Thus, there may be a slight under-estimation.

We also wanted to exclude hospitals recruited by the NTP. NTP-recruited hospitals are almost all publically funded hospitals, and IMS was able to identify and remove the data from all public hospitals. However, this may have removed some hospitals that are public but don’t collaborate with the NTP, thus leading to an underestimate of non-NTP volume.

Viet Nam

In Viet Nam, there are four major channels where patients can obtain TB therapies—public hospitals (including NTP-sponsored TB programs); pharmacies; private hospitals; and private clinics, health centers and practitioners’ offices. The IMS audit in Vietnam covered pharmacies, where 51% of all drugs (public and private) are thought to flow, but not private hospitals and clinics. Private clinics account for 10% of drug flows, although the strong reliance on dispensing private chest specialists in Viet Nam may lead to a greater than average concentration of TB drugs in this missing channel. The balance of drug flows comes from the hospital channel. In the hospital channel, less than 2% of all hospital beds are private, suggesting that any error from excluding this channel would be relatively minor. However, certain public hospitals in this excluded channel do not collaborate with the NTP and thus may have divergent treatment practices. Mitigating such a possibility is the expansion of PPM (i.e., public-public cooperation), which has increased greatly over the past 5 years. Thus, there may be an under-estimation of private market size of just over 10%.

1. Schwalbe NR, Wells WA, Geaneotes AP, Forcellina A, Lee MG, et al. (2008) Estimating the market for tuberculosis drugs in industrialized and developing nations. Int J Tuberc Lung Dis 12: 1173-1181.
